# The Role of Galectin-3 in Retinal Degeneration and Other Ocular Diseases: A Potential Novel Biomarker and Therapeutic Target

**DOI:** 10.3390/ijms242115516

**Published:** 2023-10-24

**Authors:** Ziyi Zhou, Zhaochen Feng, Xiaojia Sun, Yusheng Wang, Guorui Dou

**Affiliations:** Department of Ophthalmology, Eye Institute of Chinese PLA, Xijing Hospital, Fourth Military Medical University, Xi’an 710032, China; josiezhou_zzy@163.com (Z.Z.); 15066340726@163.com (Z.F.); sunxiaojia666@163.com (X.S.)

**Keywords:** galectin-3, retinal degeneration, molecular mechanisms, diagnostics, molecular therapy

## Abstract

Galectin-3 is the most studied member of the Galectin family, with a large range of mediation in biological activities such as cell growth, proliferation, apoptosis, differentiation, cell adhesion, and tissue repair, as well as in pathological processes such as inflammation, tissue fibrosis, and angiogenesis. As is known to all, inflammation, aberrant cell apoptosis, and neovascularization are the main pathophysiological processes in retinal degeneration and many ocular diseases. Therefore, the review aims to conclude the role of Gal3 in the retinal degeneration of various diseases as well as the occurrence and development of the diseases and discuss its molecular mechanisms according to research in systemic diseases. At the same time, we summarized the predictive role of Gal3 as a biomarker and the clinical application of its inhibitors to discuss the possibility of Gal3 as a novel target for the treatment of ocular diseases.

## 1. Introduction

Retinal diseases such as age-related macular disease, diabetic retinopathy, and glaucoma are characterized by retinal degeneration, inflammation, neovascularization, and so on [1]. Targeting key molecules in retinal degenerative disease shows its exciting potential for preventing and treating these complicated diseases. From this perspective, novel targets are urgently needed to be explored.

Galectin-3 (Gal3) is a member of b-galactoside-binding lectins weighing 32–35 kDa with a carbohydrate recognition domain (CRD) [2]. It regulates cell growth, differentiation, and inflammation, as well as many diseases related to chronic inflammation, such as neurodegeneration, cancer, fibrosis, and diabetes [3]. In the retina, the increased expression of Gal3 is related to the pathological processes of retinal degeneration, diabetic retinopathy, and retinal detachment. However, the specific role of Gal3 in retinal diseases has not been fully elucidated [4,5].

A large number of studies have confirmed that Gal3 is highly correlated with the severity of clinical manifestations of various diseases [6,7,8,9,10]. Gal3 is rapidly being used as a diagnostic or prognostic biomarker in many different clinical settings. In addition, Gal3 is involved in the progression of many diseases, especially those related to chronic inflammation, making it an effective potential drug target. Many clinical trials focusing on Gal3 are still in progress to treat serious cancers, fibrosis, metabolic diseases, and degenerative diseases. Novel inhibitors of Gal3 are under heated investigation, which engages Gal3 with excellent clinical application prospects. This review aims to summarize the pathogenesis and therapeutic prospects of Gal3 in retinal degeneration and various ocular diseases in order to promote its application in clinical treatment and bring new insights to patients suffering from visual loss.

## 2. Background of Galectins and Galectin-3

### 2.1. The Structure and Functions of the Galectin Family

**Figure 1:**
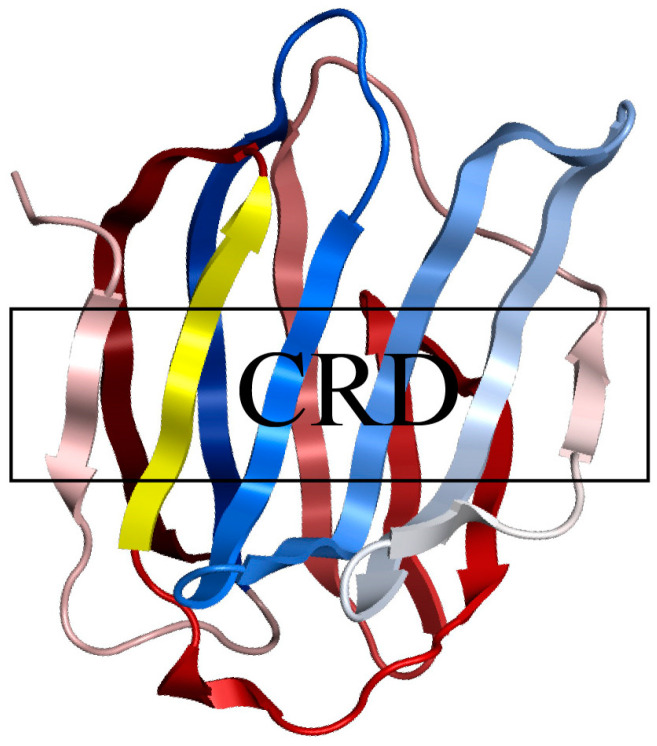
The structure of Galectin-3.

Galectins are a family composed of a highly conserved CRD characterized by the β-specificity of galactose oligosaccharides [11]. Its typical CRD sequence consists of approximately 135 amino acids, tightly folded into two 5- (or 6-) chains encoded by the LGALS gene [12] (Figure 1).

Galectins exist both inside and outside cells, and it is worth noticing that they are synthesized in the cytoplasm [13]. When they are secreted outside, a non-classical pathway is involved, which is independent of the endoplasmic reticulum or Golgi apparatus [14]. Although the exact molecular mechanisms under their secretion are not yet fully understood, their non-classical secretion seems to be influenced by IL-1β, Annexin A1, and fibroblast growth factor (FGF). Evidence showed that Galectins could be directly transported across the plasma membrane, as well as through the process driven by the Golgi apparatus posterior septum and the plasma membrane microcapsules [14]. The Galectin family may mainly be detected in the cytoplasm and nucleus, where they are stored as well as play important roles. They are involved in various biological processes, including inducing intracellular signaling, the construction of cytoskeleton and endosome tissues, and splicing nuclear RNA [15,16]. They may bind to ligands with and without carbohydrates through CRD, which exist more widely in cells and ribosomes and are related to the activation of extracellular receptors, regulating different glycolipid and glycoprotein receptors with highly distinct functions [17,18].

### 2.2. The Unique Member of Galectins—Galectin-3

Classified by structure, the Galectin family can be divided into three subtypes, including proto-type, tandem-repeat type, and chimera type [19]. Galectin-3 (Gal3) is the only chimeric one in the family. It is characterized by its evolutionarily conservative sequence with a CRD sequence at its carboxyl-terminal and a long N-terminal domain rich in proline, glycine, and tyrosine [20]. It is widely expressed in mammals and has different biological functions based on its subcellular localization. Gal3 is mainly found in the cytoplasm and nucleus and can be secreted in non-classical ways [21]. Generally, the secreted Gal3 mediates cell migration, cell adhesion, and cell-to-cell interaction through high affinity binding with glycoproteins containing galactose on the cell surface [22]. Cytoplasmic Gal3 has considerate anti-apoptotic activity and regulates multiple signal transduction pathways, while nuclear Gal3 is associated with pre-mRNA splicing and gene expression. Its unique chimeric structure enables it to interact with excessive ligands, regulating various functions such as cell growth, adhesion, migration, invasion, angiogenesis, immune function, cell apoptosis, and endocytosis, emphasizing its importance in tumor progression [13].

## 3. The Cell and Molecular Mechanisms of Gal3 in Diseases

### 3.1. Promoting Apoptosis

**Figure 2:**
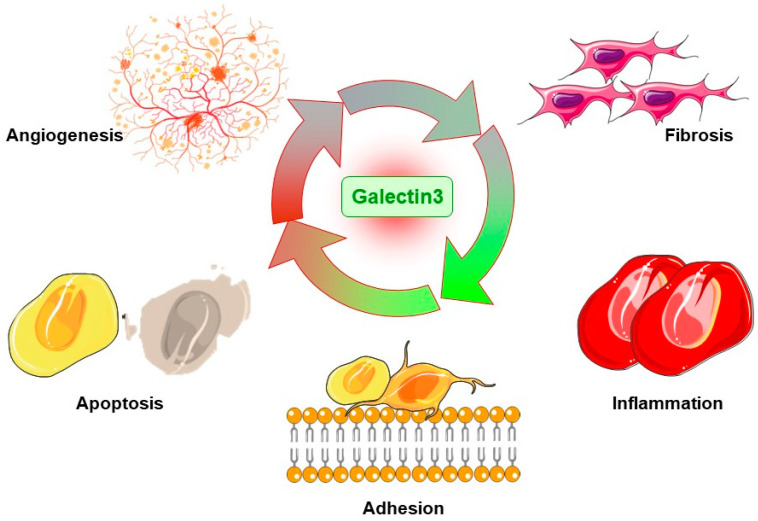
The cell and molecular mechanisms of Gal3 in diseases.

Previous studies have shown that the expression of Gal3 in human T cell lines could protect these cells from apoptosis induced by anti-Fas antibodies and broad-spectrum inhibitors of protein kinases [23]. The underlying mechanism by which Gal3 inhibits cell apoptosis is still elusive. It was shown that the function of Gal3 in apoptosis was mainly related to Bcl2 [24,25]. Bcl2 is one of the main factors in regulating apoptosis. There are similarities in the sequence between Gal3 and Bcl2 [24]. Gal3 also has the NWGR motif, which is essential for Bcl2 to regulate the activity of apoptosis and form heterodimerization with Bax [26]. In addition, Gal3 might bind to Bcl-2, which can be inhibited by lactose, indicating that CRD is involved in this interaction [24]. The importance of the NWGR motif on the apoptosis inhibitory activity of Gal3 has also been confirmed in human breast cancer cell lines. The expression of Gal3 inhibited cell apoptosis induced by cisplatin, while the expression of Gal3 with NWGR motif mutations was much less effective [27]. There is also evidence that Gal3 will transfer to the perinuclear membrane after receiving the apoptosis signal and enrich in the mitochondria to prevent mitochondrial damage and the release of cytochrome c [28]. Synexin was identified as a Gal3-binding protein that mediated this translocation. Downregulation of the expression of Synexin inhibited the translocation of Gal3 to the perinuclear membrane and weakened its ability to inhibit apoptosis [28] (Figure 2).

Gal3 may play its role via various mechanisms and interact with numerous pathways, promoting cell apoptosis, adhesion, inflammation, angiogenesis, and fibrosis.

### 3.2. Promoting Inflammation

During the development of the CNS, Gal3 is expressed in a variety of glial cells, including microglia, astrocytes, and oligodendrocytes [29], and contributes to the migration of neurocytes, myelination of oligodendrocytes, and growth of Neurite [30,31]. Meanwhile, more and more studies have proved that the Gal3 released by activated microglia in response to extra insults seems to participate in the brain immune response [32,33]. Jeon et al. demonstrated that under inflammatory conditions, such as those stimulated by IFN-γ, the expression of Gal3 in the extracellular matrix of glial cells is enhanced, and Gal3 exposure might lead to the production and upregulation of other pro-inflammatory cytokines, such as IL-1 β and IL-6, through activating the JAK-STAT and NF-κB pathways [34]. In a study using a model of cerebral ischemia and neuroinflammation, after intraperitoneal injection of lipopolysaccharide, Gal3 released by microglia was upgraded, and it played a neuroprotective and anti-inflammatory role through its CRD as an endogenous paracrine signaling ligand of TLR4 [35]. Furthermore, in the controlled cortical shock mice, it led to the downregulation of insulin-like growth factor (IGF)-1. In the models of brain injury, the upregulation of Gal3 in microglia enhanced the inflammatory response to ischemic injury [36]. In a vitro study, cerebrospinal fluid was observed 24 h after brain injury, and it was confirmed that the expression of Gal3 promoted angiogenesis and microglia migration through TLR4 transduced via the integrin-linked kinase signal [37]. Pasquini et al. reported that Gal3 promoted oligodendrocyte differentiation, aggravated ischemic damage, and neuronal apoptosis after cerebral ischemia, contributed to myelin integrity, and reduced the interaction between Gal3 and IGF-1, leading to the recovery of inflammatory demyelinating diseases [38]. The seemingly contradictory findings of downregulation of IGF-1 and inhibition of resident microglial activation might be due to different functions and locations of Gal3 in ischemic injury [36].

### 3.3. Promoting Fibrosis

Increasing evidence suggests that Gal3 is involved in promoting fibrosis in various organs, such as the liver [39], lungs [40], skin [41], kidneys [42], and heart [43]. During the process of fibrosis, Gal3 promotes the release of pro-fibrotic factors, the activation of inflammatory cells such as macrophages, and the proliferation of extracellular matrix-producing cells such as fibroblasts and myofibroblasts, thus resulting in tissue damage. In this process, Gal3 is considered to be associated with TGFβ [39,44,45]. The role of Gal3 in promoting the process of fibrosis has been confirmed in both in vitro and in vivo experiments, in which inhibiting or knocking Gal3 down may significantly alleviate fibrosis [46,47,48]. In a mouse model of renal fibrosis, Gal3 was proven to be overexpressed, while Gal3 deficiency inhibited renal fibrosis [49,50]. Also, Gal3 has been proven to be a biomarker of increased risk of heart failure and may play a crucial role in cardiac fibrosis as well [51,52]. Additionally, in vascular fibrosis, Gal3 overexpression enhances the synthesis of type I collagen in rat vascular smooth muscle cells. As the synthesis of Gal3 could be inhibited by modified citrus pectin (MCP) and specific siRNA, the synthesis of type I collagen would be downregulated; thus, the process of fibrosis would be delayed [53].

### 3.4. Promoting Adhesion

Gal3 exists in both healthy and cancer cells, as it plays a vital role in cell adhesion. It not only participates in accelerating the separation of cells from the primary tumor site but also promotes the adhesion and survival of nest-lost cancer cells in the bloodstream [54]. Cell adhesion molecule (CAM) is a membrane receptor that mediates cell–cell and cell–matrix interactions. It plays an important role in intracellular signal transduction. The interaction between Gal3 and CAM has been extensively studied [55,56,57,58]. The expression level of Gal3 is related to the biological function of integrins. Integrin is a family of heterodimeric transmembrane receptors for extracellular matrix components, such as fibrinogen (FN), laminin (LN), collagen (Col), and vitronectin (VN) [59]. Gal3 could bind to binding proteins such as LN. It also mediates cell adhesion to the ECM by regulating integrin [60]. Gal3 may regulate the migration and invasion of tumor cells by promoting the aggregation of integrin on the cell surface, mediating the endocytosis of integrin, or directly activating integrin [59,60,61]. Gal3 regulates the expression of E-cadherin through its interaction with b-catenin. Also, Gal3 co-localizes with N-cadherin and regulates the expression of N-cadherin to regulate vascular mimicry (Vm). Moreover, it binds to MUC1 through TFAG and regulates cell adhesion by exposing cadherin molecules on the cell surface. In addition, Gal3 has multiple regulatory functions on different calcium adherins. Vascular endothelial cell cadherin (VE-cadherin) is the main adhesion molecule in endothelial junctions, playing a crucial role in regulating vascular integrity and permeability. Gal3 knockout will lead to a reduction in VE-cadherin and interleukin-8 promoter activity, which will reduce the metastatic potential of melanoma cells [62,63]. Moreover, Gal3 may be involved in the nuclear preservation of b-catenin, leading to the activation of Wnt-targeted genes [64,65]. When GSK-3b activity is blocked by the Gal3/PI3K/AKT signaling pathway, the b-catenin-Gal3 complex translocates to the nucleus, binding with the TCF/Lef transcription factor family and activating the expression of target genes including c-myc, cell cyclin D1/D2, MMP7, CD44, and other TCF/Lef response genes related to cancer progression [59,66].

### 3.5. Promoting Angiogenesis

Several studies have used Gal3 knockout mice as tumor hosts to replicate the increased expression of Gal3 in tumors [67]. Gal3 has been reported to directly induce endothelial tube formation [67]. Therefore, a higher level of Gal3 promotes angiogenesis and directly affects endothelial cells within the tumor. In addition, the recruitment of Gal3+ macrophages may also be involved in the acceleration of tumor angiogenesis [67]. Gal3 promotes angiogenesis by interacting with CD146 [68], proteoglycan NG2 [69], and Notch ligand JAG1 [70]. A recent study showed that Gal3 derived from microglia could bind to endothelial Notch ligand, thereby inhibiting the activation of the Notch signaling pathway, interrupting endothelial homeostasis, and promoting retinal neovascularization [71]. In addition, Gal3 interacts with integrin through its carbohydrate recognition domain αVβ3 and then activates a certain signaling pathway to promote the growth of vessels, thereby regulating the angiogenesis mediated by VEGF and basic FGF [72]. Moreover, it could activate endothelial VEGF receptors, which might cause vascular leakage [13] and promote endothelial cell apoptosis [73], and also bring harm to blood vessels by enhancing the invasion and phagocytosis of macrophages [74]. 

## 4. The Regulation of Gal3 on Various Kinds of Retinal Cells

In retinal diseases, inflammation, autoimmune disorders, and endothelial dysfunction are usually observed. Gal3 is mainly expressed in microglia/macrophages in the retina after an inflammation insult and is also partially expressed in Müller cells [4]. According to previous studies, Gal3 might interact mainly with microglia, macrophages, and retinal pigment epithelial (RPE) cells, participating in the pathological conditions mentioned above.

### 4.1. Gal3 and Microglia

Microglia are innate immune cells in the central nervous system (CNS) that play an important role in the physiological development of the CNS and in maintaining its biological functions [75]. Gal3 is involved in enhancing microglial phagocytosis via the K-Ras-GTP and TLR pathways [31]. It regulates the phagocytosis of microglia by promoting actin/myosin-based contraction through cofilin activation and K-Ras-GTP/PI3K signaling, modulating microglial amoeboid or branched phenotypes [76]. Gal3 has complex and vital effects on microglia, which can not only promote the activation of microglia to enhance inflammation and phagocytosis but also elevate the expression of anti-inflammatory molecules from microglia, playing a neuroprotective role [77]. In recent years, a study on the delayed use of Gal3 after stroke demonstrated that the additional supplement of Gal3 increased microglial branching and mobility, altered cytokine expression profiles, and increased levels of anti-inflammatory factors such as IL-4 while decreasing the levels of pro-inflammatory factors such as TNF-α, IL-1β, IFN-γ, and IL-17 both in vivo and in vitro [78]. Gal3 plays an important role in microglial activation, especially alternative activation. It is worth noticing that Gal3 could bind to IGFR1, suggesting that the interaction between them may be necessary for IGF-mediated microglial proliferation after stroke [78]. Liu et al. indicated that Gal3 regulated microglial activation and promoted inflammation through the TLR4/MyD88/NF-κB pathway, participating in the regulation of gene expression and precursor RNA splicing in the nucleus [79]. In addition, it has been shown that Gal3 activates TREM2, enhancing phagocytosis and promoting inflammation [80].

### 4.2. Gal3 and Macrophage

The functions of invasion, phagocytosis, and pro- and anti-inflammation in macrophages have arisen to focus attention on the research of the pathological process in retinal diseases. Normal retinal tissue contains limited macrophages, while exogenous macrophages could invade the globe and participate in disease progression once the blood-retinal barrier is damaged by an extra insult [81]. There exist two activated states of macrophages, characterized by a pro-inflammatory classical activation phenotype (M1) and an anti-inflammatory alternative activation phenotype (M2) [82]. Blocking the expression of Gal3 reduced the expression of IL-8, TNF-α, and IL-1β in M1 macrophages [83]. Moreover, Gal3 takes part in the polarization of the M2 subtype, while M2 macrophages could upregulate the secretion of Gal3 in return. The Gal3 receptor, CD98, is associated with IL-4/IL-13-induced alternative activation of macrophages in vitro [84]. Gal3 could also reduce the invasion of macrophages. Gal3 was associated with the invasion of macrophages in tumors as it migrated in a Gal3-concentration-dependent manner [67]. Another study on atherosclerosis further shed light on the molecular mechanism by which Gal3 affected the invasive ability of macrophages. Through the Gal3-TGF-β1 pathway, Gal3 preserved TGF-β expression and its subsequent signaling, negatively regulating macrophage invasion, and this process could be interrupted by MMP12 [85].

Similarly, Gal3 enhances and prolongs K-Ras-GTP-PI3K-dependent signal transduction by forming complexes with K-Ras-GTP, targeting the cytoskeleton, and controlling the morphology and phagocytosis of macrophages [86,87]. Additionally, Gal3 induces MerTK activation, self-phosphorylation, and phagocytosis by binding to phagocytosis receptors of MerTK targets [88].

### 4.3. Gal3 and RPE Cells

In retinal pigment epithelial cells (RPE), Gal3 could inhibit its invasion and migration as well as protect RPE from being disturbed by an extra insult. In proliferative vitreoretinopathy (PVR), dedifferentiated RPE induced by inflammation upgrades its abilities of invasion and adhesion [89]. It has been proven that the expression of Gal3 is elevated in dedifferentiated RPE [90]. However, when RPE was incubated with Gal3 in vitro, it was observed that the spread of RPE was impaired and the abilities of invasion and adhesion in RPE were decreased, which might be partially mediated by the ERK-MAPK pathway [91]. In a model of UVA-induced retinal injury, lgals3 expression was decreased in RPE cells treated with H_2_O_2_, demonstrating that Gal3 is related to RPE cell viability, while the addition of an exogenous Gal3 supplement decreased the production of ROS and increased p38 expression, reducing oxidative stress and shedding a protective effect on RPE [92].

### 4.4. Gal3 and Endothelial Cells

Pathological retinal neovascularization and vascular leakage are the most significant changes in DR. Gal3 can directly act on endothelial cells to regulate endothelial cell migration, neovascularization, and vascular leakage, such as by binding to CD146 and integrin, which mediate its migration [56,68]. Gal3 is also involved in the regulation of endothelial cells by regulating the proteoglycan of NG2, inducing integrins by forming complexes with NG2, and promoting neovascularization [69]. At the same time, under conditions of hyperglycemia, hypoxia, and inflammation, the expression of Gal3 is upregulated, leading to enhanced VEGFR2 signaling, thereby promoting angiogenesis as well [13]. Other studies have reported that Gal3 can exacerbate endothelial dysfunction through a signaling pathway mediated by oxidized low-density lipoprotein receptor-1 (LOX-1) and may also be mediated by the LOX-1/ROS/p38/NF-κB signaling pathway, which induces inflammation and exacerbates endothelial dysfunction [93]. In an oxygen-induced retinopathy model, activated microglia upgrade Gal3, which interacts with Jag1 and blocks the Notch signaling pathway, ultimately disrupting endothelial homeostasis, improving endothelial metabolism, and promoting pathological angiogenesis [71].

### 4.5. Gal3 and Müller Cells

Müller cells are located in the inner nuclear layer (INL), with protrusions extending from the inner limiting membrane (ILM) to the outer membrane (OLM) throughout the entire retina, providing metabolic support for photoreceptors and neurons required for neural activity and maintaining their dynamic balance [94]. Müller cells can diminish fragments of retinal cells and many other foreign substances, and Gal3 can act as a MERTK ligand to activate the phagocytic function of Müller cells, thereby exacerbating retinal injury [95]. Activated Müller cells have neuroprotective effects and may participate in regulating immune and inflammatory responses under pathological conditions. They can also weaken their protective effect on neurons and exacerbate neuronal degeneration [96]. In addition to directly regulating Müller cells, Gal3 may also indirectly mediate Müller cells by affecting the activation of microglia, mediating the adaptive response after retinal injury [97]. Müller cells also release Gal3, which regulates VEGF receptors, exacerbating neovascularization and vascular leakage [13].

## 5. The Role of Gal3 in Retinal Diseases

**Figure 3:**
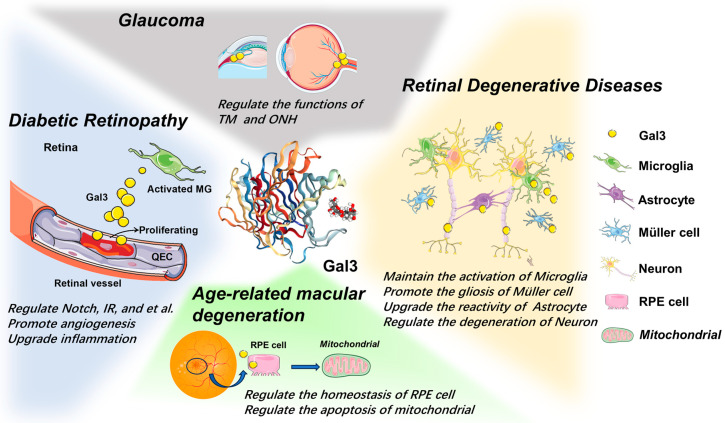
The role of Galectin-3 in ocular diseases.

Gal3 participates in the progress of several retinal diseases involving complicated mechanisms and promotes various diseases in some way (Figure 3).

### 5.1. Retinal Degenerative Disease

Gal3 is one of the mediators of microglia activation and inflammatory reactions in ischemic brain injury [36]. Previous studies have shown that Gal3 is necessary for the activation of microglia, which can promote the early inflammatory process and initiate tissue remodeling after ischemia [98]. Recently, research has confirmed that Gal3 plays a key role in the inflammation induced by synaptic nucleoprotein, which is related to Parkinson’s disease [99]. In the retina, the increased expression of Gal3 is related to the pathological processes of light-induced retinal degeneration and retinal detachment [100,101].

Gal3 plays an important role in vascular dementia [102], where the pathological process is related to the increased expression of GFAP in Müller cells as a marker of reactive gliosis after neuronal injury and to the activation and metabolic stress of microglia [103,104]. However, its mechanism has not been fully elucidated. In the brain, the progressive nerve injury after an ischemic insult is considered to be mainly due to the neuroinflammatory process, in which the production of dysfunctional cytokines shows up [105], and neurotrophin is considered to play a major role as well [106,107,108]. It has been found that after focal ischemia, Gal3 in microglia is significantly upregulated, which contributes to tissue reconstruction after ischemia [80,105]. Similarly, several retinal degenerative diseases associated with elevated GFAP and Müller cell gliosis, including light-induced retinal degeneration, are also associated with increased Gal3 expression [5,101]. Gal3 knockout significantly dampened the reactivity of glial cells and preserved photoreceptor cells and ganglion cells [71,109]. Therefore, it could be concluded that in the absence of Gal3, a mere 30% decrease in cerebral blood flow itself would not result in any significant retinal damage. The mechanism behind the activation and subsequent progress of Müller cells and microglia after ischemic injury may involve the combination and interaction of factors between these two cell types and recruited macrophages, in which Gal3 may be a key factor [110].

### 5.2. Age-Related Macular Degeneration

Age-related macular degeneration (AMD) is a progressive neurodegenerative disease in the elderly, and it is also blamed for visual impairment in the Western world [111]. Research has shown that Gal3 is significantly upregulated in the retina of patients with AMD and corresponding animal models. In activated microglia, the expression of Gal3 increases significantly, while Gal3 knockout and inhibition reduce the response of activated microglia and delay retinal degeneration [5]. Gal3 is also highly expressed in the RPE in AMD patients. Gal3, fibronectin, aggregatin, MMP2, and pigment epithelium-derived factors secreted by RPE of AMD patients are 2–3 times more than those in age-matched healthy donors [112]. Additionally, Gal3 participates in immune responses [113]. Moreover, Gal3 might regulate the intracellular homeostasis of RPE cells, regulating their growth, cell cycle, and cell death [114,115,116].

Gal3 increases the proliferation and survival ability of UVA-treated human RPE by reducing oxidative stress and increasing the activated level of p38, thus protecting them from UVA-induced cell damage [92]. Previous studies suggested that Gal3 could also regulate mitochondrial activity [28]. Gal3 postponed the opening of the mitochondrial permeability transition pore, the release of cytochrome C, and the subsequent activation of caspase-3, thus inhibiting cell apoptosis and protecting mitochondrial integrity [117,118,119]. Notably, the anti-apoptotic mechanisms of Gal3 are similar to those of Bcl2 involved in inhibiting cell apoptosis, as they share a similar sequence [27,120,121], which will be explained.

### 5.3. Glaucoma

Glaucoma is a chronic neurodegenerative disease that may result in retinal degeneration and will become a major cause of irreversible blindness worldwide by 2040 [122]. Glaucoma is related to a variety of risky factors, including glial reactivity and metabolic dysfunction of the retina and optic nerve. Although the causes are complicated, researchers mainly focus on the apoptosis of retinal ganglion cells (RGCs) and the progressive remodeling of the optic nerve head (ONH). Microglia are resident immune cells in the central nervous system (CNS) and have been identified as the key factor leading to the loss of RGC in glaucoma [123,124,125]. In the state of aging and degenerative disease, microglia switched from a quiescent phenotype to an activated one, which is characterized by the upregulation of pro-inflammatory cytokines, complements, and many other paracrine cytokines such as Gal3 [126,127]. Injecting Gal3 inhibitors into the vitreous could effectively preserve RGCs [127,128]. Meanwhile, Gal3 is elevated in the aqueous humor of glaucoma patients and is associated with a history of poorly controlled diseases [127].

There are two major ocular tissues involved in the pathogenesis of glaucoma, namely the trabecular meshwork (TM) and ONH. TM is mainly responsible for the outflow of aqueous, while ONH is the most vulnerable part in the axonal degeneration of RGCs [129]. Gal3 is expressed in the optic nerve and TM in animals [130,131]. Extracellular proteins, such as collagen, integrin, and fibronectin, have been shown to bind to Gal3 in the corneal epithelium [132]. In TM cells, Gal3 interacts with β1 integrin and induces Rho signals to regulate the adhesion and alignment of the cytoskeleton [133,134]. Furthermore, Gal3 is expressed in the uvea, corneosclera, and adjacent capillary mucosa of human TM, while no differences in expression were found between normal eyes and glaucoma [135]. Moreover, as a pathological marker of glaucoma-related chronic fibrosis, the expression of Gal3 is associated with the expression of extracellular matrix (collagen and elastin) and transforming growth factor β (TGFβ) [136]. In conclusion, it could be speculated that Gal3 is the key molecule driving the neuronal loss induced by microglia in glaucoma.

### 5.4. Diabetic Retinopathy

Diabetic retinopathy (DR) is a common microvascular complication of diabetes with systemic inflammation characterized by endothelial dysfunction and inflammation-associated retinal degeneration [137]. In the advanced stage of the disease, it is characterized by preretinal neovascularization and severe impairment of visual acuity [138]. 

In the pathological process, Gal3 was involved in the occurrence and progression of DR by triggering pathological angiogenesis, aberrant retinal metabolism, exclusive oxidative stress, and so on. The Notch signaling pathway is a key regulator in angiogenesis, and it maintains endothelial homeostasis [139]. Gal3 derived from microglia in ischemic retinopathy could competitively bind to Notch ligand to promote pathological angiogenesis [71]. In addition, elevated serum Gal3 levels in diabetic patients were associated with insulin resistance. Gal3 competitively inhibited insulin by binding to the insulin receptor (IR) and affected its downstream signals, resulting in insulin resistance; thus, an increase in the level of blood glucose could be observed, which would subsequently cause damage to retinal tissues and promote the process of diabetic retinopathy [140]. Furthermore, hyperglycemia played a destructive role through the production of advanced glycation end products (AGEs), making changes in retinal metabolism [13]. By reducing ROS production, Gal3 could reduce oxidative stress and protect retinal tissues by acting as an AGE receptor [13,92]. It was also proven that Gal3 deficiency was associated with enhanced endothelial functions [141]. It implied that Gal3 took part in DR through complicated mechanisms.

Gal3 maintains the activation of microglia, promotes the gliosis of Muller cells, upgrades the reactivity of astrocytes, and regulates the degeneration of neurons in retinal degenerative disease. Gal3 regulates the intracellular homeostasis of RPE cells, inhibits cell apoptosis, and protects mitochondrial integrity from age-related macular degeneration. In diabetic retinopathy, Gal3 promotes angiogenesis by regulating Notch and upgrades inflammation by regulating microglia, resulting in insulin resistance by binding to the IR and affecting its downstream signals. It also participates in the pathogenesis of glaucoma by regulating the functions of TM and ONH in glaucoma.

## 6. The Potential Application of Gal3 in Clinical Practice

### 6.1. Gal3 Serving as a Biomarker in Diseases

Gal3 has been widely recognized as a peripheral serum biomarker of cardiovascular diseases. A study showed that the incidence of major outcomes such as mortality, nonfatal reinfarction, stroke, and targeted vessel revascularization in patients with cardiovascular disease in the high Gal3 group was significantly higher than that in the control group. High serum Gal3 is associated with heart failure and readmission [142]. Interestingly, Asleh et al. [143] found that the increase in Gal3 was associated with an increased risk of death and heart failure in patients with acute myocardial infarction. Li et al. [144] indicated that Gal3 was an independent risk factor for major adverse cardiac events (MACEs) in patients with acute coronary syndrome (ACS). In addition, a study also pointed out that Gal3 could predict a combination of the 1-year death rate and hospitalization time for heart failure in STEMI patients [6]. The mechanisms by which Gal3 participates in cardiovascular disease and influences prognosis are still unclear, although it is recognized that Gal3 has adverse effects on cardiac remodeling and myofibroblast activation [145].

Moreover, some evidence uncovered that Gal3 promoted the inflammation of rheumatoid arthritis (RA) [146]. In collagen-induced arthritis rats, an increase in Gal3 secretion in plasma was associated with its pathological progression [147]. Compared with patients with osteoarthritis (OA) and juvenile idiopathic arthritis (JIA), the serum and synovial Gal3 levels of long-term RA patients increased significantly [148,149]. In addition, in collagen-induced arthritis rats, therapeutic administration of Gal3 shRNA might significantly inhibit the process of the disease by downregulating Gal3 expression [150]. Overall, it is suggested that Gal3 plays a crucial role in the pathogenesis of RA, and the downregulation of Gal3 may pose a new treatment strategy for RA.

In addition, Gal3 may be involved in the pathogenesis of endometriosis. The increased expression of Gal3 was detected in the peritoneal fluid of patients with endometriosis, resulting in related pain [151,152]. Gal3 is believed to be associated with myelin phagocytosis and Wallerian degeneration of neurons, as it can trigger neuronal apoptosis after nerve injury [153,154]. Gal3 is overexpressed in endometriotic lesions and may lead to neurodegeneration and unbearable pain [155].

Recently, Gal3 has been considered a potential biomarker of lung injury and a predictor of the poor prognosis of patients with COVID-19 [105,156]. More and more evidence suggests that Gal3 is involved in promoting various viral infections and enhancing pro-inflammatory cytokines such as IL-1, IL-6, and tumor necrosis factor α (TNFα) [157,158,159]. It was confirmed that Gal3 could bind to SARS-CoV-2 SPGP. Interestingly, the level of Gal3 in the blood, lung, alveolar cells, and respiratory mucus of patients with COVID-19 increased obviously [160,161]. The increased level of Gal3 in the respiratory tract of patients with COVID-19 may be related to the enhanced combination of SARS-CoV-2 and the S protein N/O chain. Gal3 may induce and promote the occurrence of acute respiratory distress syndrome (ARDS) by regulating the whole host-mediated immune sequelae of COVID-19 [161].

### 6.2. The Inhibitors of Gal3 in the Clinical Trials

The inhibitors of Gal3 have been highly discussed and have been put through clinical trials (Table 1). TD139 is a small-molecule inhibitor developed to shoot Gal3, which is mainly used as a therapy for idiopathic pulmonary fibrosis (IPF). It is characterized by nanomolecular affinity (2.3–25 nm) for Gal3 [162,163]. The safety, tolerability, pharmacokinetics, and pharmacodynamics of inhaled TD139 were evaluated through a randomized, double-blind, placebo-controlled clinical trial (NCT02257177) in phase 1/2a [164]. Inhaled TD139 was quickly absorbed and well tolerated in healthy adults and IPF patients. During the treatment, it was confirmed that TD139 had entered the target tissue by using bronchoscopy. Compared with the placebo group, Gal3 expression on alveolar macrophages decreased in the TD139 group. In addition, the inhibition of Gal3 is related to the reduction in related plasma biomarkers [164]. After this successful study, a randomized, double-blind, multicenter, parallel, placebo-controlled phase 2b clinical trial (Galaxy-1, NCT03832946) was planned to evaluate the efficacy and safety of inhaled TD139 in 426 IPF patients who received a daily dose of 3 mg for 52 weeks [164].

Another clinical trial in phase 2 of TD139 has been started (NCT04473053) to evaluate its efficacy in 200 patients with COVID-19 [165]. The purpose of this study was to investigate the delivery potential of GB0139 (another name for TD139) inhalation in patients with COVID-19 before a ventilator. It also examined whether GB0139 treatment could reduce viral load and disease severity and measured any changes in blood biomarkers. Although the trial is still in progress, the results of the pilot study on 40 patients with COVID-19 who received GB0139 plus SOC (standard nursing) and 35 patients who received only SOC treatment were recently published [165]. The combination of GB0139 and SOC presented extraordinary tolerance, indicating that GB0139 inhalation has therapeutic potential for hospitalized patients with COVID-19.

GR-MD-02 is another inhibitor of Gal3 extracted from apple pectin through chemical processing and modification [166]. It binds to both Gal1 and Gal3, while having a stronger affinity for Gal3. The study (NCT01899859) on GR-MD-02 in patients with non-alcoholic steatohepatitis (NASH) and advanced liver fibrosis (n = 31) was completed in 2015 with satisfying results in its first phase [167]. In addition, Gal3 may play a role in psoriasis. The role of Gal3 in psoriasis was discovered in the NASH clinical trial using the Gal3 inhibitor GR-MD-02. In this trial, moderate to severe plaque psoriasis was effectively treated (ClinicalTrials.gov (accessed on 19 February 2023)). Moreover, the combination of GR-MD-02 and pembrolizumab can alleviate the disease progression of patients with melanoma and non-small cell lung cancer (NCT02117362, NCT02575404, NCT04987996) [168].

### 6.3. Gal3 as a Potential Target in Ocular Disease

Gal3 is more than a biomarker indicating systematic disease; Gal3 also serves as a clue for ocular inflammation. A study focused on vernal keratoconjunctivitis (VKC) showed that a high concentration of Gal3 was detected in the tears of patients with VKC, and the concentration of Gal3 is significantly positively correlated with the degree of corneal epithelial damage [169]. A high concentration of Gal3 was detected in the supernatant of necrotic HCES, which might induce the up-expression of a variety of genes related to cell migration and cell cycle. Therefore, it is believed that Gal3 in the tears of VKC patients could serve as a biomarker reflecting the severity of epithelial damage and a potential therapeutic target [169]. 

In the treatment of retinal diseases, Gal3 has gained wide attention as well. Taking part in different cell-to-cell interactions, Gal3 promotes vitreomacular adhesion. The breakdown of Gal3 might alleviate several retinal diseases, such as macular holes related to macular traction, and has already been applied in clinical practice. As a serine protease, ocriplasmin is a truncated form of plasmin that has maintained its enzymatic properties. It is a powerful collagenase activator with proteolytic activity to break down a variety of components of the vitreoretinal interface, including Gal3. It has been approved by the European Medicines Agency for the treatment of adult vitreomacular traction and macular holes related to macular traction with a pore size ≤ 400 µm [170,171,172], implying the potential for targeting Gal3 in the treatment of retinal disease.

In our previous work, the Gal3 inhibitor significantly alleviated the progression of retinal angiogenesis by competitively inhibiting the combination of Notch1 and Jagged1, thus blocking the activation of the Notch signaling pathway [71]. As various inhibitors of Gal3 were applied in clinical trials, it is believed that Gal3 may be a promising target in ocular diseases related to both inflammation and degeneration.

## 7. Conclusions and Perspectives

With the prolongation of the lifespan worldwide, retinal diseases have become a widespread concern. Advances in molecular technologies suggest a better understanding of the pathological mechanisms of diseases and provide novel therapies in clinical practice. Considering the special immune privileges around the globe, the development of advanced medical therapies for the treatment of retinal diseases is limited and unmet. Currently, molecular therapies such as anti-VEGF drugs and steroid drugs might cause unavoidable side effects, suboptimal drug responses, and drug resistance, which indicates other promising targets should be further explored. Gal3 plays a complex role in various physiological and pathological processes. It regulates the processes of numerous diseases by inducing insulin resistance, promoting a hyperglycemic environment, and interacting with multiple cells involved in the whole body. According to a recent study, the relationship between retinal disease and systemic disease cannot be overlooked, which indicates that a drug targeting multiple processes is urgently needed [173]. Gal3 is a promising and challenging therapeutic target in retinal diseases due to its extensive cellular involvement and complexity of effects, especially in retinal diseases secondary to systemic inflammation, such as diabetic retinopathy. The combination of Gal3 and traditional drugs may be a novel direction for the treatment of ocular disease.

## Figures and Tables

**Table 1:** Gal3 inhibitors in clinical trials.

Name	Disease	Clinical Trial
TD139	idiopathic pulmonary fibrosis	NCT02257177
		NCT03832946
GB0139	COVID-19	NCT04473053
GR-MD-02	non-alcoholic steatohepatitis	NCT01899859
	non-small cell lung cancer	NCT02117362
		NCT02575404
		NCT04987996

## Data Availability

Not applicable.
